# Liver disease burden and required treatment expenditures for hepatitis C virus (HCV) infection in Thailand: Implications for HCV elimination in the new therapeutic era, a population-based study

**DOI:** 10.1371/journal.pone.0196301

**Published:** 2018-04-24

**Authors:** Rujipat Wasitthankasem, Preeyaporn Vichaiwattana, Nipaporn Siripon, Nawarat Posuwan, Chompoonut Auphimai, Sirapa Klinfueng, Napha Thanetkongtong, Viboonsak Vuthitanachot, Supapith Saiyatha, Chaiwat Thongmai, Saowakon Sochoo, Natnada Pongsuwan, Kittiyod Poovorawan, Pisit Tangkijvanich, Yong Poovorawan

**Affiliations:** 1 Center of Excellence in Clinical Virology, Faculty of Medicine, Chulalongkorn University, Bangkok, Thailand; 2 Chumpare Hospital, Chum Phae, Khon Kaen, Thailand; 3 Phetchabun Provincial Public Health Office, Mueang Phetchabun, Phetchabun, Thailand; 4 Lomkao Crown Prince Hospital, Na-saeng, Lom Kao, Phetchabun, Thailand; 5 Department of Clinical Tropical Medicine, Faculty of Tropical Medicine, Mahidol University, Bangkok, Thailand; 6 Center of Excellence in Hepatitis and Liver Cancer, Department of Biochemistry, Faculty of Medicine, Chulalongkorn University, Bangkok, Thailand; Centers for Disease Control and Prevention, UNITED STATES

## Abstract

The prevalence of hepatitis C virus (HCV) infection has been decreasing globally, but the growing effects of HCV-related morbidity and mortality remain of concern. Advances in curative medicine, involving direct-acting antivirals (DAAs), have led many countries to aim to eradicate HCV. Information on epidemiology and disease burden is essential for national policy development. Thus, this study aimed to determine the HCV-related hepatic disease burden in areas of Thailand with high and average HCV prevalence in order to extrapolate the viral burden across Thailand. Patients previously diagnosed as positive for anti-HCV antibodies were recruited to assess chronic HCV infection (CHC) status, liver function, HCV-RNA level and hepatic fibrosis. The number of patients eligible for Universal Health Coverage (UC) scheme and the approximately required expenditure on interferon (IFN)-based treatment were estimated. In areas of both high (12%) and average (2%) HCV viremic prevalence, over half of the patients (52.2% to 62.5%) had advanced liver fibrosis (F3 and F4). A striking percentage of patients with F4 (38.9%) were found in the high-prevalence area, while comparable proportions of advanced liver fibrosis presented in the two areas and disease burden peaked at 50–59 years. Under the current UC program treatment scenario, 78–83% of CHC patients with stage F2–F4 fibrosis were eligible for treatment. The estimated expenditure required for overall CHC treatment across the whole country was 1,240 million USD at this current status, but the declining cost of generic DAA-based therapy may reduce the requirement to <90 million USD. This study provides information on the estimated number of CHC patients, liver disease burden and expenditure requirements for Thailand. To eliminate HCV by 2030, proactive government strategies raising public health to minimize transmission and emphasizing targeted screen-and-treatment programs, novel therapeutic guideline development for decentralizing treatment, and effective budget allocation are urgently needed.

## Introduction

An estimated 71.1 million people are living with hepatitis C virus (HCV) infection worldwide [1]. Chronic HCV infection (CHC) is a leading cause of death due to chronic hepatitis, cirrhosis and hepatocellular carcinoma (HCC). The HCV burden accounted for 21% of the total 810,000 cancer deaths that were reported globally in 2015 [2]. Successful curative medicine, namely, direct-acting antivirals (DAAs), have shown promising results, with a >90% cure rate [3, 4]. This has raised the possibility of HCV eradication. The effective treatment has prompted the World Health Organization (WHO) to launch a Global Health Sector Strategy for viral hepatitis elimination, which aims to reduce the number of new infections and deaths by 2030 [5]. The WHO set a goal of HCV management and care by increasing the proportion of diagnosed and treated people to 90% and 80%, respectively, by 2030. To achieve this strategy, reliable information on disease burden and estimated required resources (based on epidemiological surveys by the government or other national agencies) is needed.

Of the global total, 4.7 million HCV carriers are in Southeast Asian countries including Thailand [1]. Epidemiological reports have demonstrated the declining trend of HCV viremia over the past decade [6]. The HCV seroprevalence in Thailand has decreased to 0.9%, amounting to approximately 760,000–1,475,000 and 360,000–460,000 cases of anti-HCV positive and viremia, respectively [1, 6, 7]. Since 2012, the National Health Security Office (NHSO) of Thailand has incorporated a PEGylated-interferon (PEG-IFN) based therapy for HCV into the Universal Health Coverage (UC) program. However, accessibility is likely to be limited by the screening eligibility criteria, which primarily consist of requirements related to severity and progression of liver damage.

Despite the changes in the accessibility requirements in 2014 to increase coverage, treatment still relies on IFN therapy due to the unaffordable nature of DAA-based therapy in middle-income countries. Additionally, there has been no concrete governmental surveillance response to the issue of HCV viremia. The lack of research on HCV epidemiology and related disease burden, lack of effective evaluation of the UC program and lack of robust prevention strategies has hampered the development of the HCV eradication policy at the national level.

This study aimed to determine the HCV-related disease burden in areas with high and average HCV prevalence in Thailand and to extrapolate the data to the whole country and to elucidate the proportion of HCV carriers who are eligible for the UC program according to the Thai NHSO criteria. An effective strategic plan for HCV elimination that could be implemented by the government is also discussed in this study.

## Material and methods

### Study population

**Fig 1:**
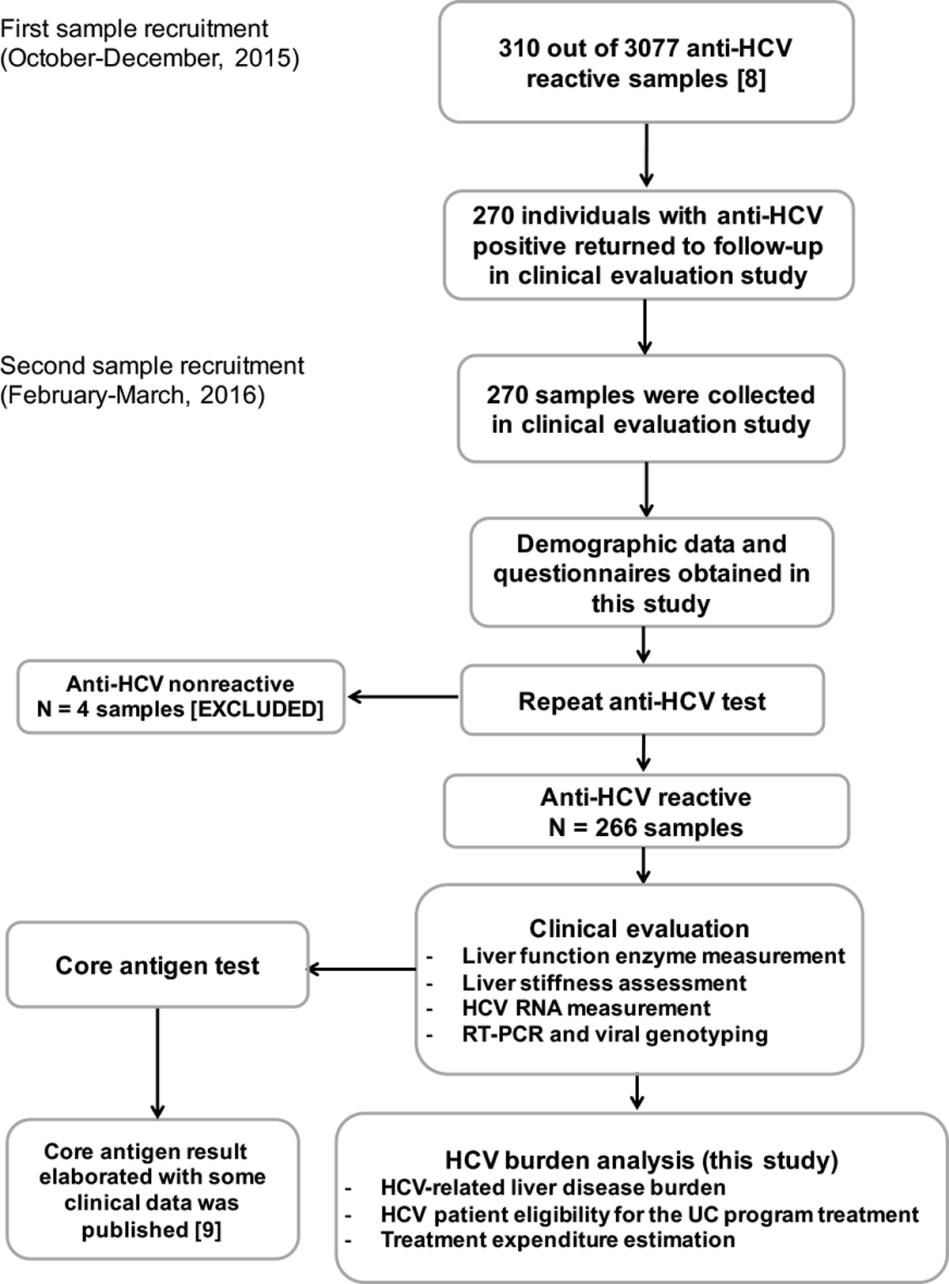
Schematic diagram of sample recruitment and clinical assessment conducted in this study.

This study was a part of an overall research project named “Prevalence and Genotypes of Hepatitis C Virus in Phetchabun and Khon Kaen Provinces as a Model for Treatment” [8]. The inclusion criteria of the overall project were: Phetchabun or Khon Kane residency, generally good health, age between 30 to 64 years, no clinical signs of immunodeficiency disease or HIV infection, and late stage of liver disease (see definition below) or hepatocellular carcinoma. The study protocol set out two independent sample recruitments (one for screening and one for clinical investigation), a serological survey [8] and follow up to clinical evaluation (Fig 1). Accordingly, most individuals who were positive for anti-HCV in the serosurvey were examined HCV related liver disease in this study.

In 2015, 3,077 individuals were recruited from Phetchabun province (a high-prevalence area) and Khon Kaen province (an average-prevalence area) in Thailand. They were screened for HCV serological markers. All anti-HCV antibody-positive samples were tested for HCV-RNA by reverse transcription-polymerase chain reaction (RT-PCR) [8]. In March 2016, the participants with positive serological anti-HCV results were all informed of their infection status and invited to follow-up in this clinical assessment including anti-HCV retesting, liver enzymes determination, liver stiffness evaluation, HCV RNA measurement and HCV genotyping by RT-PCR. Of the total, 270 individuals (231 and 39 from Phetchabun and Khon Kaen, respectively) with anti-HCV antibody-positive have recruited in this study (Fig 1). Demographic data and related risk factors were collected from all participants.

The study protocol was approved by the Institutional Review Board of the Faculty of Medicine, Chulalongkorn University (IRB no. 258/58). Each subject was informed of the study objectives, and written consent was obtained from all subjects.

### Laboratory tests

To assess HCV-associated liver injury severity, the subjects were tested for clinical parameters including re-testing for anti-HCV antibodies (ARCHITECT, Abbott Diagnostics, Wiesbaden, Germany), liver enzymes (aspartate transaminase [AST] and alanine transaminase [ALT]) and plasma HCV-RNA level (Abbott RealTime HCV assay, Abbott Molecular, IL, USA). Hepatic fibrosis was evaluated by transient elastography (TE; FibroScan, Echosens, Paris, France). HCV genotyping was performed by nucleotide sequencing. Laboratory tests in this study were previously mentioned in a published literature base on HCV core antigen test (Fig 1) [9]. Serological screening markers for hepatitis B virus surface antigen (HBsAg) and human immunodeficiency virus (HIV Ag/Ab) were previously tested and included to further analysis in this study [8].

### Fibrosis stage evaluation

Hepatic fibrosis staging was used to analyze the proportion of individuals with the HCV-related liver disease. Liver stiffness was evaluated using the METAVIR scoring system, F0–1, F2, F3 and F4, according to TE values ≤7.0, >7.1–9.5, >9.5–12.5 and >12.5 kPa, respectively [10]. Advanced liver disease was defined as an individual with naïve chronic hepatitis C and significant fibrotic stage (TE >9.5 kPa or METAVIR score ≥F3) while definition of advanced liver disease was one of clinically symptoms of decompensated liver cirrhosis (jaundice, hepatic encephalopathy, clinically detectable ascites and variceal bleeding) and/or hepatocellular carcinoma which was excluded in the epidemiological surveillance step [11].

### UC reimbursement and required expenditure

A subgroup analysis of the individuals who met the Thai NHSO criteria for UC reimbursement, covering for HCV screening, treatment, and monitoring, was carried out. Thai patients meeting the following criteria were eligible for treatment under the UC program: aged 18–65 years, hepatic fibrosis with TE value ≥7.5 kPa (or METAVIR score ≥F2), plasma HCV-RNA level ≥5,000 IU/mL, no sign of decompensated liver illness and not active HIV co-infection (Table 1). AST and ALT levels >30 IU/mL were assumed to represent elevated liver function enzymes. The expenditures required for HCV screening, treatment and monitoring were based on the median cost of government hospitals and the Thai Food and Drug Administration (FDA) medicine prices (~95 USD per week, http://drug.fda.moph.go.th:81/nlem.in.th/medicine-price; accessed 3 Nov 2017), which are indicated in S1 Table. The estimated expenditures were calculated based on genotypic proportions (genotype 1, 3 and 6), the approximate number of HCV-infected patients in each of the two areas and the cost per treatment of the respective genotype (genotypic proportion * infected population *cost of treatment). An estimate for the whole of Thailand was calculated based on the fibrosis severity in the average-prevalence area (in this study). The size of the anti-HCV positive and CHC population in Phetchabun, Khon Kaen and Thailand were an age-stratified estimation and retrieved from previous studies [6,8]. The length and treatment cost of PEG-IFN plus ribavirin-based treatments for genotypes 1, and 6 is 48 weeks, and 4,560 USD per course, for genotype 3 is 24 weeks and 2,280 USD per course, respectively [12]. The length of DAAs treatment regimen for all genotype is 12 weeks, and generic drug pricing of sofosbuvir was estimated at 250 USD per course [3,13].

**Table 1:** National Health Security Office (NHSO) criteria for HCV treatment with Peg-IFN plus ribavirin.

	Year 2012–2014	Year 2014 to present
**Age range (year)**	18 to 65	18 to 65
**Treatment experience**	Naïve	Naïve
**Stage of liver disease exclusion**	End stage	End stage
**Alcohol consumption**	Stop drinking ≥ 6 months	Stop drinking ≥ 6 months
**Liver function test**		
**AST**	≥ 1.5 fold of normal level	NR
**ALT**	≥ 1.5 fold of normal level	NR
**Genotype**	genotype 2 and 3	genotype 1,2,3 and 6
**HCV viral load**	≥ 5,000 IU/mL	≥ 5,000 IU/mL
**Liver stiffness**^a^ **(Kpa)**	≥ 7.5	≥ 7.5
**HIV status**	No infection	Viral load not detected
**CD4**	NR	> 350^b^ or > 500^c^ cell/mL

^a^Transient elastography

^b^Under HAART treatment

^c^No HAART treatment

NR: Not required

### Data analysis

Chi-squared tests and one-way analysis of variance (ANOVA) with a Bonferroni correction were used to evaluate the difference between groups and group means, respectively (AST, ALT, age and viral RNA level). All statistical analyses were performed using SPSS for Windows (version 11.5; SPSS, Chicago, IL, USA).

## Results

**Fig 2:**
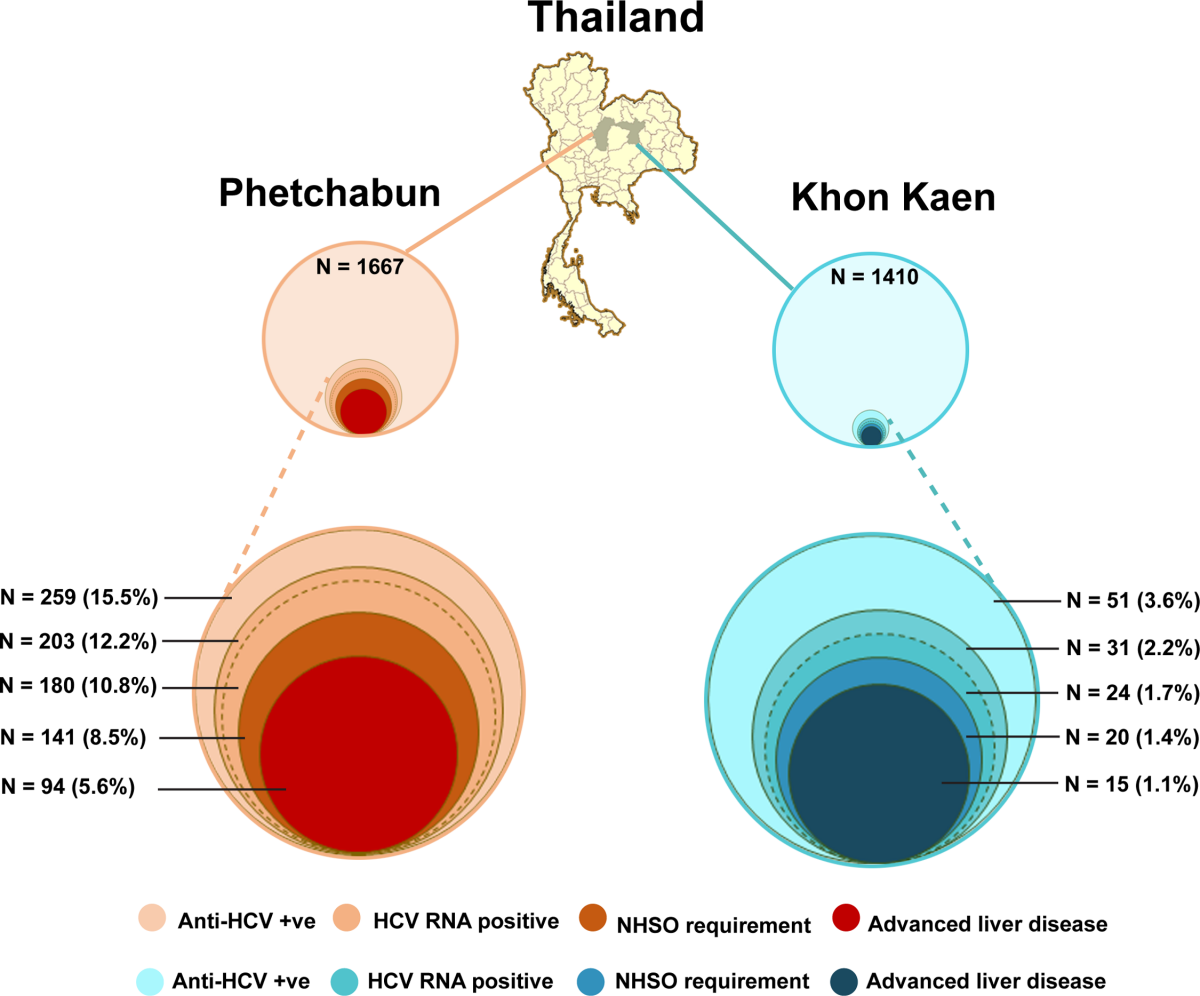
HCV epidemiology and disease burden in areas with high and moderate HCV prevalence in Thailand. Samples recruited from Phetchabun (n = 1667) and Khon Kaen (n = 1410) were screened for anti-HCV antibodies (upper panel). Among all anti-HCV antibody-positive individuals, HCV RNA positive (RNA positive sample in the follow-up study is indicated in a dashed circle), eligibility for universal coverage (according to the NHSO requirements) and hepatic fibrosis were all evaluated (lower panel). The NHSO criteria for HCV treatment reimbursement were TE ≥7.5 kPa and viral load ≥5000 IU/mL. The advanced liver disease was represented by liver stiffness >9.5 kPa or a fibrosis score (METAVIR stage F3-F4). The proportion of individuals in each category is indicated by the size of the circle diagrams.

This study involved a previous HCV epidemiological surveillance and a present clinical evaluation study that investigated HCV infection prevalence and liver disease burden in two adjacent provinces, Phetchabun and Khon Kaen (Fig 1). During the previous surveillance step, Phetchabun (a high-prevalence area) had 15.5% of participants with anti-HCV antibodies and 12.2% with viremia (Fig 2). Khon Kaen (an adjoining province with an average prevalence) had much lower prevalence of anti-HCV antibodies (3.6%), and only 2.2% were HCV-RNA-positive. In these two areas, HCV was predominant in males, and the most common genotype was genotype 6 (Table 2, upper panel). Co-infection with hepatitis B virus (HBV) was 3.0% in HCV viremic samples (7/234).

**Table 2:** Epidemiological and clinical characteristic of HCV infection in Phetchabun and Khon Kaen province.

	HCV prevalence		
	High	Average	Total
	Phetchabun	Khon Kaen	
**HCV surveillance study**			
**Number of samples**	1,667	1,410	3,077
**M/F (ratio)**	774/893 (0.9:1)	556/854 (0.6:1)	1,330/1,747 (0.8:1)
**Anti-HCV +ve (%)**^a^	259 (15.5)	51 (3.6)	310 (10.1)
**M/F (ratio)**	220/39 (5.6:1)	42/9 (4.7:1)	262/48 (5.5:1)
**HBsAg +ve (%)**^b^	13 (5.0)	2 (3.9)	15 (4.8)
**HCV RNA +ve (%)**^b^	203 (78.4)	31 (60.8)	234 (75.5)
**M/F (ratio)**	180/23 (7.8:1)	26/5 (5.2:1)	206/28 (7.4:1)
**HCV RNA & HBsAg +ve(%)**^c^	6 (3.0)	1 (3.2)	7 (3.0)
**Genotype (%)**^c^			
**1**	64 (31.5)	10 (32.3)	74 (31.6)
**3**	64 (31.5)	9 (29.0)	73 (31.2)
**6**	75 (37.0)	12 (38.7)	87 (37.2)
**HCV follow-up to clinical evaluation study**			
**Number of samples**	231	39	270
**Anti-HCV +ve (%)**^a^	228 (13.7)	38 (2.7)	266 (8.6)
**HCV RNA +ve (%)**^d^	180 (78.9)	24 (63.2)	204 (76.7)
**HIV Ag/Ab +ve (%)**^e^	1 (0.6)^f^	0 (0.0)	1 (0.5)
**Liver stiffness (%)**^e^			
**F0-1 (≤ 7 kPa)**	31 (17.2)	2 (8.3)	33 (16.2)
**F2 (>7.0 kPa)**	54 (30.0)	7 (29.2)	61 (29.9)
**F3 (> 9.5 kPa)**	24 (13.3)	7 (29.2)	31 (15.2)
**F4 (>12.5 kPa)**	70 (38.9)	8 (33.3)	78 (38.2)
**ND**^g^	1 (0.6)	0 (0.0)	1 (0.5)
**≥ 7.5 Kpa (%)**	148 (82.2)	22 (91.7)	170 (83.3)
**Viral load (%)**^e^			
**≥ 5000 IU/mL**	173 (96.1)	22 (91.7)	195 (95.6)
**NHSO requirement (%)**^e^			
**Liver stiffness** **≥** **7.5 and viral load** **≥** **5000 IU/mL**	141 (78.3)	20 (83.3)	161 (78.9)

^a^Percentage calculated based on total sample in surveillance study

^b^Percentage calculated based on anti-HCV positive in surveillance study

^c^Percentage calculated based on HCV RNA positive in surveillance study

^d^Percentage calculated based on anti-HCV positive in clinical study

^e^Percentage calculated based on HCV RNA positive in clinical study

^f^One sample with triple HCV/HBV/HIV infection

^g^One sample of Phetchabun cannot determine

In this clinical evaluation study, 228 and 38 individuals from Phetchabun and Khon Kaen, respectively, were confirmed to be anti-HCV antibody-positive (Table 2, lower panel). In total, HCV-RNA was found in 76.7% (204/266) samples by RT-PCR and RNA measurement. Only one sample was HCV/HIV co-infection (0.5%, 1/204). Liver function enzymes (AST and ALT) were significantly elevated in CHC individuals (S2 Table) and half of them (52.2–62.5%) had advanced liver disease (F3–4, Table 2, lower panel). There was no significant difference in mean age and viral load between different liver stiffness stage groups, but there were significant differences in liver function enzyme levels. Patients with a fibrosis stage F4 had significantly elevated AST and ALT levels compared with the other groups (*p* < 0.001 and *p* = 0.001, respectively, S3 Table).

**Fig 3:**
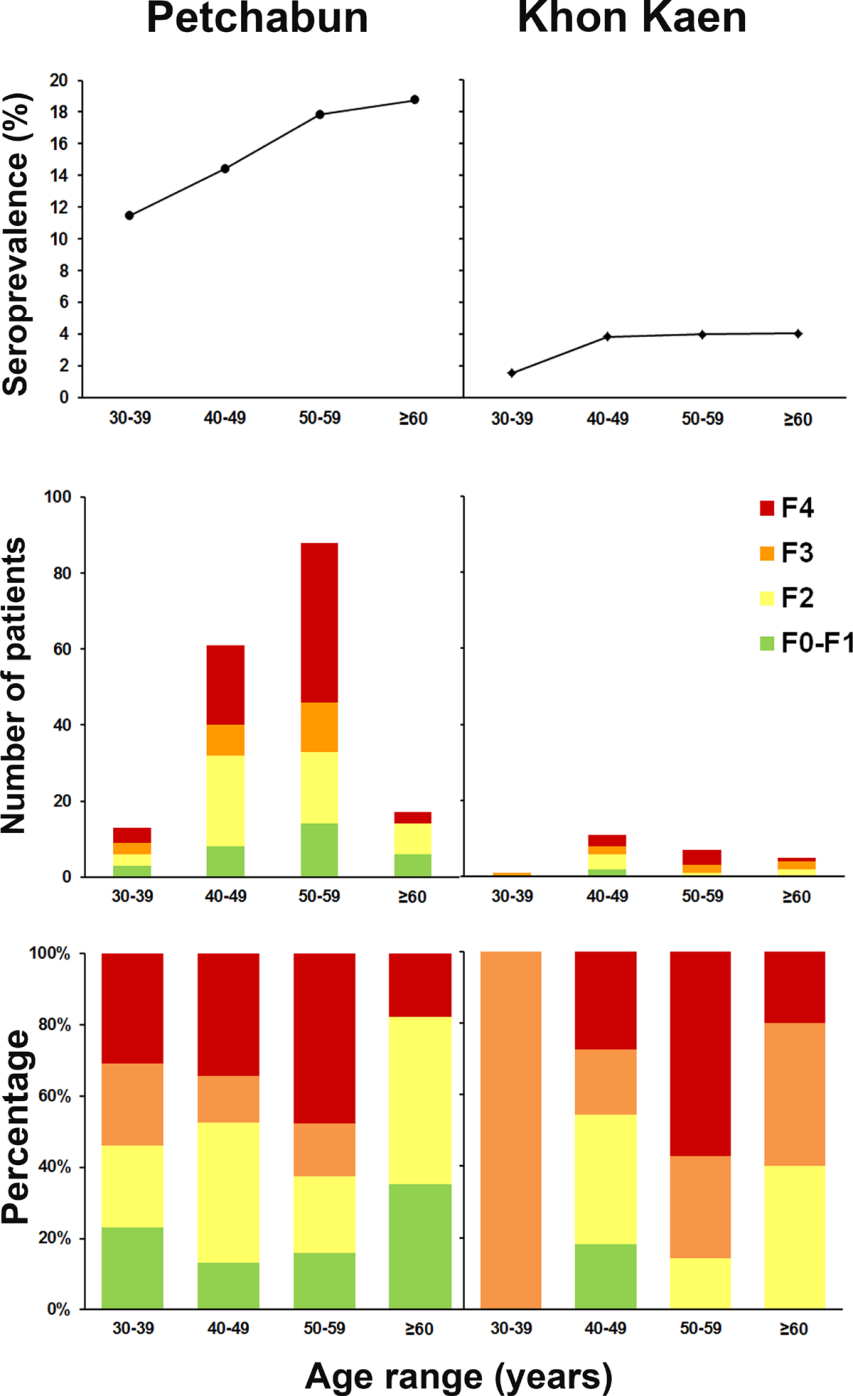
Liver fibrosis stages and age group in areas with high (Phetchabun) and average (Khon Kaen) HCV prevalence in Thailand. Seroprevalence of HCV is indicated in the upper panel. The number of patients (middle panel) and the percentage of patients (lower panel) for each hepatic fibrosis stage are shown according to each age group in Phetchabun (left panel) and Khon Kaen (right panel).

Fig 2 shows the differences in the proportions of HCV carriers between the two areas (percentage was calculated with respect to the total samples in surveillance study). The seroprevalence and viremic rate in Phetchabun were approximately four and six times higher than the rates in Khon Kaen, respectively, leading to a six-fold higher number of patients with advanced liver disease in the former province than the later (Table 2). The advanced hepatic fibrosis severity peaked at age 50–59 years and decreased in the older age group (Fig 3). Overall, 83.3% (170/204) of CHC patients were TE values ≥7.5 kPa while 95.6% (195/204) had a viral load ≥5000 IU/mL. The proportion of patients who were eligible for the UC program (based on a combination of these two parameters, according to the NHSO criteria) was 78.9% (161/204).

Table 3 shows the population size, prevalence of anti-HCV antibodies and prevalence of viremic carriers, as well as the CHC individuals with F3-F4 stage in Phetchabun and Khon Kaen provinces. The approximate numbers of CHC individuals with the advanced liver disease were 6,072 (5,773:100,000 population) individuals in Phetchabun province and 581 (1,390:100,000 population) individuals in Khon Kaen province. The disease burden of the whole nation could not be estimated due to lack of representative information on the population aged <30 years. Regarding the whole country, 356,670 individuals had active HCV infection, of whom 343,698 were aged >30 years. By applying data from the average-prevalence area, the CHC burden who aged >30 years was estimated to be 214,811 (576:100,000 population) individuals with the advanced liver disease.

**Table 3:** Estimated budget required for interferon-based HCV treatment in Thailand.

	Phetchabun (high endemic)	Khon Kaen (average endemic)	Thailand	
**Age of determination**	30 to 64 years^a^	30 to 64 years^a^	31 years and above^b^	6 months to 71 years^b^
**Population number**	105,171^a^	41,818^a^	37,308,533^c^	63,954,350^b^
**Sampling population number**	1,667^a^	1,410 ^a^	2,290^c^	5,964^b^
**Estimated anti-HCV carrier (%)**^d^	14,998 (14.3)^a^	1,432 (3.4)^a^	635,159 (1.7)^c^	758,940 (1.2)^b^
**1:100000**	14,261^a^	3,424^a^	1,702^c^	1,187^b^
**Estimated HCV RNA carriers (%)**^d^	11,633 (11.1)^a^	930 (2.2) ^a^	343,698 (0.9)^c^	356,670 (0.6)^b^
**1:100000**	11,061^a^	2,224^a^	921^c^	558^b^
**Advanced liver disease (F3-F4)**	6,072^e^	581^e^	214,811^f^	ND
**1:100000**	5,773^e^	1,390^e^	576^f^	ND
**Genotypic proportion**				
**Genotype 1**	31.5%^a^	32.2%^a^	17.4%^b^	
**Genotype 3**	31.5%^a^	29.0%^a^	47.8%^b^	
**Genotype 6**	37.0%^a^	38.7% ^a^	34.8%^b^	
**Estimated treatment budget (USD)**^g^^,^^h^				
**Genotype 1**	16,974,874	1,357,056	272,703,741	282,996,245
**Genotype 3**	8,487,437	614,916	374,575,828	388,713,233
**Genotype 6**	19,627,198	1,653,912	545,407,482	565,992,490
**Total**	45,089,509	3,625,884	1,192,687,051	1,237,701,968

^a^data derived from previous study [8]

^b^data derived from previous study [6]

^c^data derived from individuals aged ≥31 reported from a previous study [6]

^d^Prevalence and percentage were an age-stratified estimation and retrieved from the previous studies [6,8]

^e^Data calculated from HCV RNA carriers in Phetchabun or Khon Kaen [8] and the respective percentage of advanced liver disease found in this study

^f^Data calculated from HCV RNA carriers aged ≥31 and percentage of advanced liver disease speculated from Khon Kaen province in this study

^g^Currency exchange rate at 20/11/2017 = 33 Baht to 1 USD (https://www.bot.or.th/Thai/Pages/default.aspx)

^h^Treatment expenditure was estimated based on the total HCV RNA carrier*genotypic proportion*treatment cost

The Thai FDA has set the median price for HCV traditional treatment (PEG-IFN plus ribavirin) leading to expenditure per treatment course of 4,560 USD for genotypes 1 and 6, and 2,280 USD for genotype 3 (S1 Table). Due to unavailable pricing of DAAs and unstable novel treatment regimen in Thailand, the approximate cost relied on the Indian generic sofosbuvir pricing at 250 USD per treatment cost [13]. Therefore, the estimated expenditure required for HCV carriers (30–64 years) in Phetchabun and Khon Kaen by traditional treatment is 45,089,509 and 3,625,884 USD and by novel therapy is 2,908,250 USD and 232,500 USD, respectively (Table 3). For the whole nation, the approximately-required expenditure of the PEG-IFN based therapy is 1,237,701,968 and 1,192,687,051 USD for the total CHC population and those aged >30 years, respectively, while the novel treatment expenditure is approximately required 89,167,500 USD and 85,924,500 USD for the total CHC and those age >30 years, respectively.

## Discussion

Regarding transfusion-transmitted and blood-borne infections, Thai public health has been improving, as evidenced by the low prevalence of HCV infection during 2004–2014 [6, 14, 15]. Since the implementation of HCV screening in 1992, the seroprevalence in the general population has decreased to 0.9% [6]. However, rates of complicated liver diseases and CHC-related death are expected to increase in the coming decades due to the progression of diseases [16]. The lack of information on disease burden is an obstacle to establishing effective policies for prevention, screening and HCV elimination on a national scale [17]. This study provides evidence-based data on CHC-related hepatic fibrosis that shows most of the patients had progressed to advanced disease stages.

A previous epidemiological study demonstrated that HCV prevalence was dramatically different in two adjoining provinces in Thailand (Phetchabun and Khon Kaen), and it showed that a history of intravenous drug abuse and tattooing contributed to the significant risk of HCV infection [8]. The present study evaluated liver fibrosis among those HCV carriers from the previous study population [8], in both high- and average-prevalence areas (Phetchabun and Khon Kaen), and found that most had CHC and progression to liver fibrosis (≥F2). As expected, there were a high number of advanced liver disease patients (≥F3) in the high-prevalence area, but disease severity proportion in both areas seems to be comparable in all age group (Fig 3). A striking number of patients with the advanced disease stage (52.5–62.5%) were at risk of developing HCC and complications. This burden rose with age and peaked at 50–59 years. The progression of liver fibrosis has been shown to be non-linear but significantly consistent with the duration of infection [18]. The probability of progression of liver fibrosis to cirrhosis (F4) at 20 and 30 years after the infection has been shown to be 16% (range, 7–18%), and 41% (range, 36–45%), respectively [18]. It seems that the accumulation of patients with advanced liver fibrosis in the 50–59 age group may have been due to these patients acquiring the infection >20–30 years ago, which is consistent with the period before national implementation of HCV screening and high levels of intravenous drug abuse in the study areas [8]. In contrast, individuals aged ≥60 years had a lower prevalence of advanced liver diseases, which may be related to later HCV acquisition than those in the younger age group.

An estimated 356,670 Thai individuals live with CHC and most (96%) are aged >30 years (approximately 343,698), which is the age at which HCV prevalence rises [6]. Among the people with CHC, approximately 214,811 individuals (aged >30) may develop advanced liver fibrosis. These populations have a risk of rapid progression to severe disease, including HCC. Fortunately, current treatment allocation in Thailand prioritizes advanced liver disease stages [16]. We found that 78–83% of HCV patients (covered CHC patients with liver fibrosis stages F2–F4) in this study met the NHSO criteria and were eligible for HCV treatment via the UC program. Although achieving a sustained virologic response (SVR) to treatment can reduce HCV-related mortality, patients with cirrhosis are still at risk of ongoing HCC development (0.3–2.4% annual incidence) and hepatic decompensation over the years [19–21]. In addition to initial urgent treatment, HCC surveillance of cirrhotic patients after obtaining an SVR should be maintained during long-term follow-up appointments [19, 20].

The disease burden and serious complications due to HCV infection are projected to increase over the next few decades [22, 23]. It is expected that liver disease complications will increase by three- to four-fold under the current treatment scenario, even in countries with a very low viremic prevalence (0.2–0.5% among adults) [24]. Although effective IFN-free DAA treatment can improve the therapeutic response (>90% with SVR), the cumulative death due to CHC depends on the treatment coverage and allocation strategies [16]. In Norway, increasing the coverage of DAA treatment to 50% is speculated to be able to reduce the incidence of cirrhosis by half [22], while, in Iran, doubling or stepwise increase in the current coverage is expected to reduce the number of deaths by >60% by 2030 [23]. Similar projections have been calculated for Thailand, in that the current cumulative HCV-related death will diminish (25.5% reduction), and CHC prevalent will decline to nearly zero under expanded treatment strategy (DAA based-therapy) by increasing in fivefold increments of the current coverage (3,000 treatments in 2015) over the next 20 years [16].

Thailand has just received a voluntary license for generic DAAs from Gilead Sciences, the drug company. According to the estimated number of CHC patients in the Thai population [6], under the current IFN-based treatment scenario, approximately 1,240 million USD is required for the overall treatment cost (2280–4560 USD per course) or 103 million USD per year (for 12 years) up to 2030. However, these numbers may be overestimated because of the declining price of the original DAAs and the cost of generic drugs, which may come down to ≤250 USD per treatment course [13]. If so, the total expenditure needed for HCV treatment will decrease to 89 million USD overall or 7.4 million USD per year.

While preparing this study, the Thai Ministry of Public Health issued a strategic plan for HCV elimination that involved, from the beginning of 2018, using sofosbuvir in the PEG-IFN plus ribavirin regimen for patients with HCV genotype 3 and an IFN-free DAA regimen for patients with non-genotype 3, and from the beginning of 2020, an IFN-free DAA regimen for all patients. The Ministry of Public Health has set up a strategic plan for HCV elimination in the first 5 years after 2017. The foremost objective is to increase treatment coverage to 50% and then to decrease viral transmission via extensive blood donor screening and harm reduction approaches [25]. The program includes five strategies: 1) disease surveillance and database system development; 2) prevention, harm reduction and increase in infection awareness; 3) patient recruitment and care; 4) translating scientific research into disease prevention and control strategies; and 5) effective resource and budget allocation.

Nevertheless, a challenge to the government is to recruit the CHC patients, as asymptomatic cases and mild clinical signs represent a barrier. As HCV therapeutic costs decline over time, expensive screening methods may not be necessary (an estimated cost for HCV screening and linkage to care per case was 260 USD, S1 Table). Thus, to increase access and the number of diagnosed people, simplifying the diagnostic algorithm to produce a low-cost algorithm by supplementing the HCV core antigen (HCVcAg) test (instead of the nucleic acid test [NAT]) after a seropositive test could be key, especially in high-prevalence settings [9, 26, 27].

Moreover, targeted screening would enhance the success of HCV elimination. Together with our results, we propose that targeted screening should involve: 1) high-risk groups such as intravenous drug users, 2) people in high-prevalence areas such as Phetchabun province, 3) people in high-risk age groups (either 50–59 or ≥30 years); and 4) encouraging the normal population to undergo HCV screening during annual check-ups or routine screening during blood donation.

The limited number of specialist physicians may obstruct the accessibility of CHC treatment. However, the highly effective and well-tolerated DAA therapy has raised the possibility of task shifting to the primary care setting to expand rapid access to treatment, offer comprehensive care and engage patients with treatment adherence programs. In Pakistan and Columbia, primary care management of CHC patients was shown to result in positive outcomes (83% with SVR) [28, 29]. Non-specialist providers (such as nurse practitioners and primary care physicians) were shown to be able to administer safe and effective HCV treatment with as high a cure rate as specialists, though loss to follow-up was found to be a major cause of non-SVR [29]. Thus, the development of guidelines and health care training would be essential approaches to generalize and decentralize the HCV treatment strategy.

This study had several limitations. Liver fibrosis related to alcohol consumption could not be determined and taken into account. The infection acquisition time points could not be extrapolated due to unclear information obtained from the participants. Cirrhotic stages could not be assessed. The liver fibrosis status and HCV treatment expenditure respective to the whole country may be overestimated due to the lack of representative information and was estimated based on the disease burden found in Khon Kaen where the HCV seroprevalence rate was slightly higher than that in the country.

This study provided information on liver fibrosis severity in areas with high and average HCV prevalence. The advanced liver fibrosis peaked at similar age group (50–59) in the two areas. We provided information on the number of patients who were eligible for UC program coverage (based on NHSO criteria, which covered almost all CHC individuals with progressing disease stages), the expenditure needed for overall treatment with traditional therapy and the estimated number of CHC patients with the advanced liver disease. Such information will help Thailand to estimate the required internal and external sources of funding in order to allocate budgets effectively. Approaches to restructuring the eligibility criteria and encouraging the target population to obtain treatment should be carefully developed by the Thai Ministry of Public Health. Research should be conducted on the optimal resource and budget allocation across different diseases and the expected benefits (reflecting impacts on public health and the economy) in order to effectively improve welfare and the overall development of the population.

## Supporting information

S1 Table Laboratory and treatment expenditures required for HCV therapy and expenditure provided by the Thai government. (DOCX)

S2 Table Liver function enzymes and HCV RNA status. (DOCX)

S3 Table Clinical parameters and hepatic fibrosis among patients recruited from Phetchabun and Khon Kaen. (DOCX)
