# Supplementary material for: Liver disease burden and required treatment expenditures for hepatitis C virus (HCV) infection in Thailand: Implications for HCV elimination in the new therapeutic era, a population-based study
Source: PLoS One. 2018 Apr 24;13(4):e0196301. doi: 10.1371/journal.pone.0196301 (PMC5916520; doi:10.1371/journal.pone.0196301)
Supplement: S1 Table — (DOCX) [file pone.0196301.s001.docx]

**S1 Table.** **Laboratory and treatment expenditures required for HCV therapy and budget supported by the government.**

| **HCV screening** | |
| --- | --- |
| **Test** | **Cost per test (USD)^a^** |
| SGOT (AST) | 2 |
| SGPT (ALT) | 2 |
| CBC^b^ | 3 |
| Anti-HCV | 9 |
| HBsAg | 4 |
| Anti-HIV | 4 |
| HCV viral load | 70 |
| Genotyping | 120 |
| Transient elastography | 45 |
| Total (real cost) | 260 |
|  |  |
| The UC reimbursement^c^ | 210 |
| **HCV Treatment^c^** | |
| **Treatment cost^d^ (weeks)** | **Cost per individual (USD)^a^** |
| Genotype 1 (48) | 4560 |
| Genotype 3 (24) | 2280 |
| Genotype 6 (48) | 4560 |
| **Treatment monitoring^b^** | |
| **Viral load measurement^e^ (times)** | **Cost per individual (USD)^a^** |
| Genotype 1 (4) | 180 |
| Genotype 3 (2) | 90 |
| Genotype 6 (4) | 180 |

^a^Currency exchange rate at 20/11/2017 = 33 Baht to 1 USD (https://www.bot.or.th/Thai/Pages/default.aspx).

^b^Complete blood count

^c^Budget cover by UC program.

^d^Treatment cost supported by the program equal to ~ 95 USD per week.

^e^Viral load measurement cost equal to ~ 45 USD per time.
