# Supplementary material for: Liver disease burden and required treatment expenditures for hepatitis C virus (HCV) infection in Thailand: Implications for HCV elimination in the new therapeutic era, a population-based study
Source: PLoS One. 2018 Apr 24;13(4):e0196301. doi: 10.1371/journal.pone.0196301 (PMC5916520; doi:10.1371/journal.pone.0196301)
Supplement: S2 Table — (DOCX) [file pone.0196301.s002.docx]

**S2 Table. Liver function enzymes and HCV RNA status**

|  |  |  | **AST** |  | **ALT** |  |
| --- | --- | --- | --- | --- | --- | --- |
|  | **HCV RNA** | **N** | **Mean (SD)** | **p** | **Mean (SD)** | **p** |
| **Phetchabun** | **negative** | 48 | 27.9 (23.3) | 0.000 | 19.6 (17.0) | 0.000 |
|  | **positive** | 180 | 64.0 (57.2) |  | 50.9 (51.3) |  |
| **Khon Kaen** | **negative** | 14 | 25.1 (8.0) | 0.000 | 19.7 (9.0) | 0.001 |
|  | **positive** | 24 | 62.8 (35.5) |  | 58.5 (39.4) |  |
