# Supplementary material for: Liver disease burden and required treatment expenditures for hepatitis C virus (HCV) infection in Thailand: Implications for HCV elimination in the new therapeutic era, a population-based study
Source: PLoS One. 2018 Apr 24;13(4):e0196301. doi: 10.1371/journal.pone.0196301 (PMC5916520; doi:10.1371/journal.pone.0196301)
Supplement: S3 Table — (DOCX) [file pone.0196301.s003.docx]

**S3 Table. Clinical parameters and hepatic fibrosis of patients recruited from Phetchabun and Khon Kaen.**

|  |  | **F0-1 (≤ 7 kPa)** | **F2 (>7.0 kPa)** | **F3 (> 9.5 kPa)** | **F4 (>12.5 kPa)** | **Total** | **p** |
| --- | --- | --- | --- | --- | --- | --- | --- |
| **Phetchabun** | **N** | 31 (17.3%) | 54(30.2%) | 24 (13.4%) | 70 (39.1%) | 179 | ND |
| **Mean (SD)** | **Age** | 50.9 (7.6) | 50.1 (7.6) | 49.2 (7.5) | 51.0 (6.2) | 50.5 (7.0) | 0.696 |
|  | **AST** | 37.1 (21.8) | 45.5 (32.8) | 52.7 (46.0) | 94.5 (71.9) | 64.2 (57.3) | < 0.001 |
|  | **ALT** | 32.5 (21.5) | 38.3 (30.1) | 58.3 (79.0) | 66.7 (57.3) | 51.1 (51.4) | 0.002 |
|  | **Log HCV RNA** | 6.3 (0.8) | 6.0 (1.0) | 5.8 (1.1) | 5.9 (1.1) | 5.9 (1.0) | 0.204 |
| **Khon Kaen** | **N** | 2 (8.3%) | 7 (29.2%) | 7 (29.2%) | 8 (33.3%) | 24 | ND |
| **Mean (SD)** | **Age** | 46.0 (0.0) | 51.0 (6.7) | 51.6 (10.3) | 51.5 (7.1) | 50.9 (7.5) | 0.834 |
|  | **AST** | 31.5 (6.4) | 54.1 (33.2) | 54.3 (24.9) | 85.5 (40.5) | 62.8 (35.5) | 0.123 |
|  | **ALT** | 26.0 (17.0) | 58.1 (44.8) | 46.9 (41.2) | 77.0 (32.6) | 58.5 (39.4) | 0.304 |
|  | **Log HCV RNA** | 6.9 (0.1) | 6.0 (1.0) | 5.6 (1.3) | 5.5 (1.1) | 5.8 (1.1) | 0.453 |
| **Total** | **N** | 33 (16.3%) | 61 (30.0%) | 31 (15.3%) | 78 (38.4%) | 203 | 0.197 |
| **Mean (SD)** | **Age** | 50.6 (7.5) | 50.2 (7.5) | 49.7 (8.1) | 51.1 (6.2) | 50.5 (7.1) | 0.813 |
|  | **AST** | 36.8 (21.2) | 46.5 (32.6) | 53.1 (41.8) | 93.6 (69.2) | 64.0 (55.1) | <0.001 |
|  | **ALT** | 32.1 (21.1) | 40.5 (32.3) | 55.7 (71.8) | 67.7 (55.2) | 51.9 (50.1) | 0.001 |
|  | **Log HCV RNA** | 6.3 (0.7) | 6.0 (1.0) | 5.7 (1.1) | 5.8 (1.1) | 5.9 (1.0) | 0.078 |
